# *kernInt*: A Kernel Framework for Integrating Supervised and Unsupervised Analyses in Spatio-Temporal Metagenomic Datasets

**DOI:** 10.3389/fmicb.2021.609048

**Published:** 2021-01-28

**Authors:** Elies Ramon, Lluís Belanche-Muñoz, Francesc Molist, Raquel Quintanilla, Miguel Perez-Enciso, Yuliaxis Ramayo-Caldas

**Affiliations:** ^1^Plant and Animal Genomics, Statistical and Population Genomics Group, CSIC-IRTA-UAB-UB Consortium, Centre for Research in Agricultural Genomics (CRAG), Bellaterra, Spain; ^2^Department of Computer Science, Polytechnic University of Catalonia, Barcelona, Spain; ^3^Schothorst Feed Research B.V., Lelystad, Netherlands; ^4^Animal Breeding and Genetics Program, IRTA, Caldes de Montbui, Spain; ^5^Catalan Institution for Research and Advanced Studies (ICREA), Barcelona, Spain

**Keywords:** microbiome, metagenomics, kernel, supervised, unsupervised, spatio-temporal, SVM, kPCA

## Abstract

The advent of next-generation sequencing technologies allowed relative quantification of microbiome communities and their spatial and temporal variation. In recent years, supervised learning (i.e., prediction of a phenotype of interest) from taxonomic abundances has become increasingly common in the microbiome field. However, a gap exists between supervised and classical unsupervised analyses, based on computing ecological dissimilarities for visualization or clustering. Despite this, both approaches face common challenges, like the compositional nature of next-generation sequencing data or the integration of the spatial and temporal dimensions. Here we propose a kernel framework to place on a common ground the unsupervised and supervised microbiome analyses, including the retrieval of microbial signatures (taxa importances). We define two compositional kernels (Aitchison-RBF and compositional linear) and discuss how to transform non-compositional beta-dissimilarity measures into kernels. Spatial data is integrated with multiple kernel learning, while longitudinal data is evaluated by specific kernels. We illustrate our framework through a single point soil dataset, a human dataset with a spatial component, and a previously unpublished longitudinal dataset concerning pig production. The proposed framework and the case studies are freely available in the *kernInt* package at https://github.com/elies-ramon/kernInt.

## Introduction

The microbiome is defined as the ensemble of microorganisms and their genomes in a given environment. Microorganisms are present in ecological niches as diverse as soil, oceans, freshwater, plants, and animals, but a large fraction of these taxa cannot be cultivated with culture-dependent methods. The advent of next-generation sequencing (NGS) revolutionized this field by allowing the massive sequencing and quantification of microbial habitats.

Proper analysis of microbiome data is challenging for a variety of reasons. Abundance data obtained with NGS is multivariate, sparse and compositional in nature (Gloor et al., 2017). Also, microbial communities are very dynamic biological systems, thus justifying spatial or time-course studies (Bodein et al., 2019; Berg et al., 2020). The first approach on the field used statistical tools from standard ecological studies (Gloor et al., 2017). For example, one of the first steps in nearly all microbiome studies consists in computing alpha and beta-diversities. Beta-diversity measures, e.g., Bray-Curtis or Unifrac, quantify the difference in diversity between samples from different habitats. They are used for clustering analysis or, more commonly, for visualization techniques like principal coordinates analysis (PCoA) or multidimensional scaling (MDS). However, this approach has been challenged, as the abundance data obtained by NGS has a particular nature. The total number of reads delivered is bounded by an uninformative sum: the library size (i.e., the number of total reads per sample). Library size is uninformative because it does not contain information about the population. Instead, it is arbitrarily fixed by the sequencing process and may vary by orders of magnitude across samples (McMurdie and Holmes, 2014). This kind of data is called compositional and deserves a specific mathematical treatment (Gloor et al., 2017). In the case of metagenomics, extensive research is being done to translate current statistical techniques to this paradigm (Gloor et al., 2017; Silverman et al., 2017; Rivera-Pinto et al., 2018). One example is the proposal of using the compositional Aitchison distance instead of the classic beta-diversity measures (Quinn et al., 2018).

In machine learning, the aforementioned clustering, ordination and visualization techniques belong to the so-called unsupervised learning. Supervised learning, which is focused on prediction, is not so widespread in microbiome analysis yet, but the number of studies using this kind of approach is rapidly growing in the last years (Zhou and Gallins, 2019). Due to this rise in popularity, widely used libraries for microbiome analysis like QIIME2 (Bolyen et al., 2019) now include plugins for supervised learning in their toolbox. Typical available methods include random forests (RF), artificial neural networks (ANN), support vector machines (SVM), and ridge regression (Qu et al., 2019; Zhou and Gallins, 2019; Namkung, 2020). Among the aforementioned, RF are popular in the microbiome context and tend to outperform other methods (Zhou and Gallins, 2019; Namkung, 2020). ANN have shown excellent performance in some cases but are susceptible to overfitting, especially if sample size is greatly exceeded by the number of taxa, as is often the case in metagenomics and metataxonomics. A desirable feature for supervised methods is the identification of microbial signatures (i.e., taxa that are predictive of a certain phenotype), which may enable a biological interpretation of the results. RF are endowed with variable importance measures that can be used to this effect, while there is not such straightforward heuristic for ANN, although several possible strategies exist (Ibrahim, 2013). Another supervised method, *selbal* (Rivera-Pinto et al., 2018), is focused on the identification of microbial signatures based on balances (i.e., the geometric means of data from two groups of taxa), and has the particularity of being purely compositional.

As microbial communities are highly dynamic systems, it is important to address their spatial and/or temporal variation (Berg et al., 2020). In spatial-structured studies, repeated samples of different sites (e.g., body sites, depth layers) are obtained from the same individuals or entities, thus raising the question of how to integrate them. A more general challenge is the integration of datasets coming from different sources (e.g., “omics”), which may have different data types. Several statistical methods have been proposed to solve this question in the microbiome field. Some examples are *Link-HD* (Zingaretti et al., 2020), *mixKernel* (Mariette et al., 2018), and *MOFA* (Argelaguet et al., 2018), all focused in the unsupervised learning setting. In most supervised methods, this integration is usually performed at the input data level (early integration), for example by concatenating the datasets; or after the model is built (late integration), combining their scores as in ensemble methods. However, early integration may be not possible if data nature differs across sources (Schölkopf et al., 2004). The case of the longitudinal studies (which follow the evolution over time of microbial communities) is more complex. Typically, longitudinal data is modeled by fitting a function (e.g., polynomial interpolation, splines) to the data points over time. To date, there exist few analytical tools for this kind of data in the microbiome field. Two examples can be found at Bodein et al. (2019) and Coenen et al. (2020), but they are restricted to unsupervised analysis.

**FIGURE 1:**
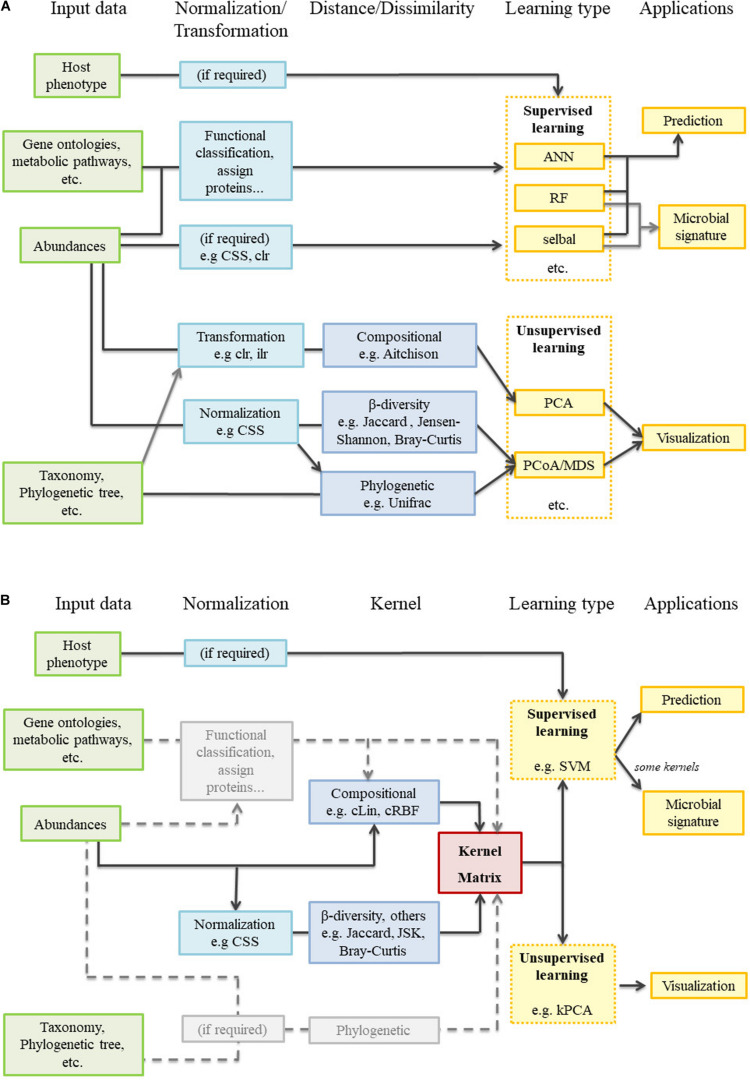
Metagenomic analysis workflow. **(A)** Current state-of-the art: supervised and unsupervised learning are completely independent. **(B)** Kernel framework: the pivotal position of the kernel matrix is clearly observed. In gray, several tasks not performed during the present work but that merit future research.

Difficulties like the compositionality of data or how to accommodate the spatial and temporal dimensions affect supervised and unsupervised methods alike. However, there is a gap between the most widely used supervised learning methods and the unsupervised analyses typical of the microbiome field (Figure 1A). Libraries like QIIME2 juxtapose traditional analyses (e.g., PCoA) with many different and powerful prediction algorithms, but both branches remain independent from a mathematical point of view. It is true that some beta-diversity dissimilarity-based engines can be used as classifiers (Su et al., 2020; Shenhav et al., 2019). However, as these tools are strongly focused on distinguishing among a limited number of bacteria-related conditions, they are not aimed at regression problems, nor do they give any information about the microbial signatures. We consider that carrying out all aforementioned analyses in a common mathematical framework would provide a new, holistic view to microbiome studies. With all this in mind we propose a generic and flexible kernel framework (Figure 1B) as a way to handle unsupervised and supervised microbiome analyses (including the retrieval of microbial signatures), while paying special attention to data compositionality and spatial and temporal integration. Kernel methods are a family within machine learning methods that share the use of kernel functions or, simply, kernels. Some of these methods have been already applied to some specific problems or areas within microbiome analysis (Zhan et al., 2017; Mariette et al., 2018; Zhou and Gallins, 2019) but their potential has not been fully exploited. In this work, we propose two new compositional kernels and discuss how to translate non-compositional, but nonetheless widespread, beta-diversity matrices to the kernel framework. We perform supervised and unsupervised analyses from the same kernel matrix, and show how to extract microbial signatures. Spatial and longitudinal data are also treated with specific kernel tools. This kernel framework is illustrated with three case studies: a single point soil metagenomic dataset, a human dataset with a spatial component, and a previously unpublished longitudinal dataset concerning pig gut microbiota. An R package implementing the proposed methods, along with the analyzed datasets, is freely available at https://github.com/elies-ramon/kernInt.

## Materials and Methods

### Kernels for Microbiome Data

A real symmetric two-place function is a kernel iff, for every finite set of objects *x*_1_,…,*x*_*N*_, it generates a positive semi-definite matrix of dimension *N*×*N*: the kernel matrix (Schölkopf et al., 2004; Shawe-Taylor and Cristianini, 2004). Probably the most widely known and used kernel functions are the linear and radial basis function (RBF) kernels, both defined for real vectors.

Intuitively, a kernel can be understood as a measure of the similarity between *x*_*i*_ and *x*_*j*_. As objects *x*_1_,…,*x*_*N*_ are never represented explicitly, kernels can be designed for non-standard data types if a notion of what is considered “similar” in that given context exists (Schölkopf et al., 2004). Each kernel provides a different grasp of the dataset. Furthermore, as similarity measures, kernels are related (but opposite) to the beta-diversities widely used in microbiome analyses. However, although every beta-diversity distance or dissimilarity is paired with a similarity counterpart, not all of them fulfill the aforementioned conditions and are, therefore, kernels.

We now present two compositional and two non-compositional kernels, all of them available in *kernInt*. In addition, users have the option of entering any kernel matrix, pre-computed with a kernel of their choice. In this work we are restricted only to kernels that can be obtained from taxonomic abundance tables, but further insights can be found in the Discussion.

#### Compositional Kernels

Here we define two kernels analogous to the linear and RBF kernels, but specific for compositional data. We introduce the Aitchison-RBF kernel as:

(1) $$cRBF(x_{i},x_{j})\;=exp(-\gamma \sum_{k=1}^{D}{\left(log\left(\frac{x_{ik}}{G\left({\text{x}}_{\text{i}}\right)}\right)-\;log\left(\frac{x_{jk}}{G\left({\text{x}}_{\text{j}}\right)}\right)\right)}^{2})$$

where *x*_*i*_ and *x*_*j*_ represent the taxonomic abundances in two different individuals, *D* is the number of different taxa, *G(.)* is the geometric mean, and γ > 0 is a hyperparameter that has to be tuned. This non-linear kernel derives from the Aitchison distance, which is Euclidean and therefore (Eq. 1) is a valid kernel. The logarithm term can be identified as the compositional clr-transformation (Gloor et al., 2017) over the original data.

Analogously, we define the compositional linear kernel as:

(2) $$c\,L\,i\,n\,\left({\text{x}}_{i},{\text{x}}_{j}\right)=\sum_{k=1}^{D}l\,o\,g\,\left(\frac{x_{i\,k}}{G\,\left({\text{x}}_{\text{i}}\right)}\right)\,l\,o\,g\,\left(\frac{x_{j\,k}}{G\,\left({\text{x}}_{\text{j}}\right)}\right)$$

Although cRBF is related to Aitchison distance and has the advantage of non-linearity, cLin is easier to interpret and allows the retrieval of the microbial signatures.

#### Non-compositional Kernels

The most widely beta-diversity measures are Bray-Curtis, Unifrac and Jensen-Shannon (Gloor et al., 2017). Bray-Curtis and Jensen-Shannon are computed from taxonomic tables, while Unifrac additionally needs a phylogenetic tree. The Jensen-Shannon is metric and has a kernel counterpart that is already described in Bai and Hancock (2011) as the Jensen-Shannon Kernel (JSK):

(3) $$JSK\left({\text{x}}_{i},{\text{x}}_{j}\right)=1-\frac{1}{2}[\sum_{k=1}^{D}x_{i\,k}\ln\left(\frac{2\,x_{i\,k}}{x_{i\,k}+x_{j\,k}}\right)+\sum_{k=1}^{D}x_{j\,k}ln\left(\frac{2\,x_{j\,k}}{x_{i\,k}+x_{j\,k}}\right)]$$

provided that *x*_*i*_ and *x*_*j*_ contain relative frequencies. The Bray-Curtis dissimilarity is semimetric, and so we propose using Jaccard, a similar distance (Gardener, 2014), instead. The Jaccard distance is paired with a well-known kernel (Bouchard et al., 2013) and has a variant suitable for quantitative data. The quantitative Jaccard (also known as Ružička) kernel is defined in Gardener (2014) as:

(4) $$q\,J\,a\,c\,\left({\text{x}}_{i},{\text{x}}_{j}\right)=\sum_{k=1}^{D}\frac{m\,i\,n\,\left(x_{i\,k},x_{j\,k}\right)}{m\,a\,x\,\left(x_{i\,k},x_{j\,k}\right)}$$

All aforementioned kernels have an asymptotic computational complexity of *O*(*N*^2^*D*).

#### Kernel Methods and Framework

Kernel methods share the use of symmetric and positive semi-definite matrices (i.e., kernel matrices), and not the original data, as input. That limits the potential similarity measures that one can use to only valid kernels, but also guarantees that every matrix generated can be processed by the kernel method. Furthermore, using kernels places all different analyses in a common mathematical ground (see Figure 1B), which we refer as the kernel framework. For phenotype prediction, we use SVM, a classical method that can perform regression and classification (both binary and multi-class). For the unsupervised analyses we use kernel principal components analysis (kPCA), a kernelized version of the standard algorithm. In both cases, *kernInt* allows the user to choose the values of the hyperparameters and (in the case of SVM) to perform a complete cross-validation and performance evaluation using an independent test set.

### Spatial Data

The kernel framework is particularly well suited for the integration of spatial or heterogeneous data types (Schölkopf et al., 2004; Mariette et al., 2018). This is because the integration can be done directly at the kernel matrices level. Let _**K1**_,…,_**KM**_ be the kernel matrices computed from *M* different sources of data coming from the same individuals. Then, we can obtain a consensus kernel matrix ^K*^:

(5) $${\text{K}}^{*}=\sum_{z=1}^{M}\beta_{z}\,{\text{K}}_{\text{z}}$$

with the restriction β_*z*_≥0. The optimal β_*z*_ values can be obtained through an optimization process, which is known as multiple kernel learning (MKL) (Schölkopf et al., 2004). In unsupervised scenarios, a consensus matrix ^K*^ can be obtained by choosing the β coefficients that maximize average similarity of ^K*^ with all _**Kz**_ matrices (Mariette et al., 2018).

### Temporal Data

A time series is an ordered set of repeated samples indexed by time, in the form {*x*_*i*_,*t*_*i*_}. The natural way to summarize this type of data is through a function, which can be obtained using polynomial interpolation or splines. When data contains the time series of several individuals, it is commonly referred as longitudinal data.

The functional RBF kernel (Chen et al., 2013) translates the RBF kernel to accept real functions as input. Therefore, evolution over time among individuals is compared and used afterward for phenotype prediction or unsupervised tasks. Let *f*(*t*) and *g*(*t*) be univariate functions, so that they represent the variation of a single feature in two different individuals between the time interval [*t*_*a*_, *t*_*b*_]. Then, the kernel definition is:

(6) $$f\,R\,B\,F\,\left(f,g\right)=e\,x\,p\,\left(-\gamma \,\int_{t_{a}}^{t_{b}}{\left|f\,\left(t\right)-g\,\left(t\right)\right|}^{2}\,d\,t\right)$$

In an analogous way, the functional linear kernel is defined as:

(7) $$f\,L\,i\,n\,\left(f,g\right)=\int_{t_{a}\,}^{t_{b}}f\,\left(t\right)\,g\,\left(t\right)\,d\,t$$

These kernels allow irregular sampling intervals and missing time points, but suffer of the cost of computing numerically the integral (e.g., if an algebraic solution is not possible). Computations can be simplified if fLin and fRBF are defined for discrete functions, so the modeling of time series as continuous functions is skipped. In this case, *f*(*t*) and *g*(*t*) may directly denote the original objects {*x*_*i*_,*t*_*i*_}, so each time value directly maps to a certain value of the feature variable *x*. If *T* is the total number of time points and Δ*t* the time increment, then:

(8) $$f\,R\,B\,F\,\left(f,g\right)=e\,x\,p\,\left(-\gamma \,\sum_{i=1}^{T}{\left(f\,\left(t_{i}\right)-g\,\left(t_{i}\right)\right)}^{2}\right)$$

(9) $$f\,L\,i\,n\,\left(f,g\right)=\text{Δ}\,t\,\sum_{i=1}^{T}f\,\left(t_{i}\right)\,g\,\left(t_{i}\right)$$

The discrete approach is sound in cases with few data points, when the modeling is less reliable. However, contrarily to (Eqs 6, 7), these expressions cannot deal with irregular sampling times or missing data.

In multivariate scenarios, for instance microbiome data, many features are simultaneously sampled over time. Let *f*_*k*_ and *g*_*k*_ model taxon *k* in two individuals, being *D* the total number of taxa. The aforementioned kernels can be combined as in:

(10) $$f\,R\,B\,F^{\prime }\,\left(f,g\right)=\prod_{k=1}^{D}f\,R\,B\,F\,\left(f_{k},g_{k}\right)$$

(11) $$f\,L\,i\,n^{\prime }\,\left(f,g\right)=\sum_{k=1}^{D}f\,L\,i\,n\,\left(f_{k},g_{k}\right)$$

With a computational complexity of *O*(*N*^2^*TD*) if (Eqs 8, 9) are used.

It should be noted that the kernel approach allows the integration of data that is both spatial and temporal-structured. *kernInt* first handles the temporal dimension using a kernel for longitudinal data (fLin or fRBF) over each space point, and then integrates the spatial dimension by performing MKL over the fLin or fRBF kernel matrices coming from the same individual.

### Microbial Signature

In a broad sense, the “microbial signature” is the collection of taxa associated with a trait of interest that has a high predictive value in the context of a given model (Rivera-Pinto et al., 2018). It can be retrieved from a linear SVM using the orientation of the separating hyperplane (Guyon et al., 2002): if the plane is orthogonal to a particular feature dimension, then that feature is maximally informative. This method takes into account the correlation between taxa. As cLin is a translation of the linear kernel for compositional data, using (Eq. 2) we can retrieve the microbial signatures, which should be understood as the taxa importances after the clr-transformation. The same occurs when assessing the variable influence on the principal components in kPCA. A general permutation technique is proposed in Mariette et al. (2018), but using cLin permits obtaining the taxa influence in the same straightforward way than standard principal components analysis (PCA).

The linearity also permits extending the microbial signature retrieval, when using SVM, to the longitudinal and spatial cases. When performing MKL, as long as the cLin kernel is strictly applied to all sampled sites, the global importance of a given taxon among all sites can be computed as the weighted sum (using the optimal β coefficients) of its partial importance in each site. In the longitudinal case, the global importance of each taxon *k* can be obtained from (Eq. 9) by addition of the partial importances over all *T* time points.

### Case Studies and Data Pre-processing

We illustrate our framework with three case studies: a single point dataset, a dataset with a spatial component, and a longitudinal dataset. The latter is previously unpublished while the rest of the data is public.

#### Soil Dataset

Bacterial composition of soil varies significantly at a biogeographical scale, and is related to chemical and environmental factors. Here we reanalyzed a single point dataset by Lauber et al. (2009), who used 16S small-subunit ribosomal (16S rRNA) gene pyrosequencing to profile the bacterial communities of different soils across North and South America. Authors reported that soil pH was significantly correlated with beta-diversity distances between samples. They also found correlation with alpha diversity, which was highest in soils with near-neutral pHs. To perform our analysis, we retrieved the taxonomic abundances as well as the associated metadata from Qiita https://qiita.ucsd.edu/ (ID: 103). The number of operational taxonomic unit (OTUs) was 7,396, while the number of soil samples was 89. As a part of the pre-processing, we excluded sample number 89, with only 1 read, which was also not included in the original paper.

#### Smokers Dataset

Charlson et al. (2010) analyzed the impact of cigarette smoking on the global airway microbial population. Bacterial communities were profiled using 454 pyrosequencing of the 16S rRNA gene in four airway sites: the left and right sides of nasopharynx and oropharynx. Authors reported that composition was primarily determined by airway site, with individuals exhibiting minimal lateral or temporal variation. They used RF to predict the smoking status from the taxonomic abundances. We retrieved the dataset (metadata and taxonomic abundances) from Qiita (ID: 524) to perform our analysis. Of the original 70 individuals, we discarded those that reported airway illness or antibiotic usage in the 3 months prior to sampling. Thus, we analyzed the same 62 individuals of the original work (29 smokers and 33 non-smokers). Number of different OTUs was 2,817.

#### Pig Dataset

Here we present a previously unpublished dataset, which evaluates the relationship of pre-weaning diarrhea with the early gut microbiota colonization in piglets. Gut microbiota was profiled in 153 piglets during their first week of life. Between days 8 and 21 (weaning day), 79 out of the 153 piglets had diarrhea and were treated with antibiotics. Swab sampling was done within 5 min after farrowing (day 0) and at days 3 and 7 post-farrowing. DNA was extracted from fecal samples and profiled using Illumina sequencing of 16S rRNA gene in each of the three time points. The cleaned sequences were processed into amplicon sequence variants (ASVs). Further details are described in Supplementary Method 0. Analyses were carried out at the ASV (3,577 ASVs were obtained) and at the Genera taxonomic levels.

### Experimental Set-Up

Analyses across the three datasets included a comparison with the original reports (for Soil and Smokers datasets), as well as contrast with results from RF. The cLin and cRBF kernels were applied directly to the raw counts, as they handle data in an inherently compositional manner. Before computing both kernels, a number under the detection limit was added to all dataset entries to handle zeroes (Quinn et al., 2018). An alternative normalization of data, the cumulative sum scaling (Paulson et al., 2013) was performed prior to applying the non-compositional Jensen-Shannon and Jaccard kernels. That way the compositional and non-compositional kernels could be compared. In the rest of cases (RF and longitudinal) we used the compositional clr-transform over data. RF were obtained with the R package *randomForest* (Liaw and Wiener, 2002), while the kernel approach was carried out using *kernInt* (which relies on the *kernlab* package for computing kPCA and SVM). A step-by-step guide with examples can be found at the *kernInt* package vignette: https://elies-ramon.github.io/kernInt/.

Unsupervised analyses were carried out using the whole datasets. Instead, for the supervised analyses, each dataset was split at random into the training set (80% of data) and the test set (20%). Optimal hyperparameters’ values (number of trees in RF, cost in SVM, and γ for RBF-like kernels) and β coefficients for MKL were obtained by 5 × 5 cross-validation on the training set. Hyperparameters’ ranges are in Supplementary Table 1. Once the best values were found, the final model was built using the whole training set. We repeated the whole process 40 times, each time with different 80/20 randomly split training/test partitions, to obtain an error distribution. Performance over the test set was computed using normalized mean squared error (NMSE) for regression and Accuracy for classification. We measured with the *microbenchmark* package the running time of computing the SVM models on a 64-bit Ubuntu 20.04 LTS workstation with Intel(R) Core(TM) i5-6300U CPU at 2.40 GHz and 12 GiB of RAM (see Supplementary Figure 1). For the sake of comparison, the running time of several RF implementations (including the *randomForest* package) can be found at Wright and Ziegler (2017).

For the Smokers and Pig case studies, additional considerations had to be taken into account. In the Smokers dataset, in addition to the kPCA analysis, we computed the similarity among kernel matrices of different body sites with the *mixKernel* package (Mariette et al., 2018). We compared the performance of data integration via MKL (the kernel approach) with that of RF when using early and late integration approaches. In the former case, the input of the RF was the concatenated data of the four sites. Instead, in the latter case we used the forests created for each site separately to vote for the final decision (Li et al., 2018).

In the Pig dataset, to make sure that the training and test sets were completely independent, piglets from the same litter (full sibs) were always placed either in one or other set. Performance of fLin and fRBF was contrasted to those of RF and their analogous non-longitudinal kernels (cLin and cRBF) using all available days at once. For the non-longitudinal methods, 80% of the piglets were used to train the model, using their three time points data in separate rows, with time included as an additional variable. The remaining piglets were reserved to test the model, but using only one of their time points (either day 0, 3, or 7) chosen at random and discarding the rest. This way, both longitudinal and non-longitudinal approaches had the same test set size. Longitudinal kernels fLin and fRBF were computed using (Eq. 9) and (Eq. 8), as only three time points were available and we preferred not to interpolate the day’s in-between. Also, using the expression for discrete functions we could obtain the microbial signatures. The information of all taxa was combined as in (Eqs 11, 10) and the training/test partitions were carried out as in the normal case. In a second step, the dataset was decomposed by sampling times and the analysis was carried out for days 0, 3, and 7 separately using RF, cLin and cRBF in the usual way.

Microbial signatures from SVMs were obtained from the hyperplane normal vector *w*. The importance of taxon *k* is computed by *kernInt* as ^(*wk*)2^ (Guyon et al., 2002). When using RF, we used the mean decrease in node impurity (for regression tasks) and mean decrease in Gini index (for classification). Both RF and SVM give absolute values of taxa importance, so they were converted to relative values. We used the R package *MiRKAT* (Zhan et al., 2017) to test if the association of the target phenotype with the signatures we obtained was statistically significant.

## Results

### Soil Data

**FIGURE 2:**
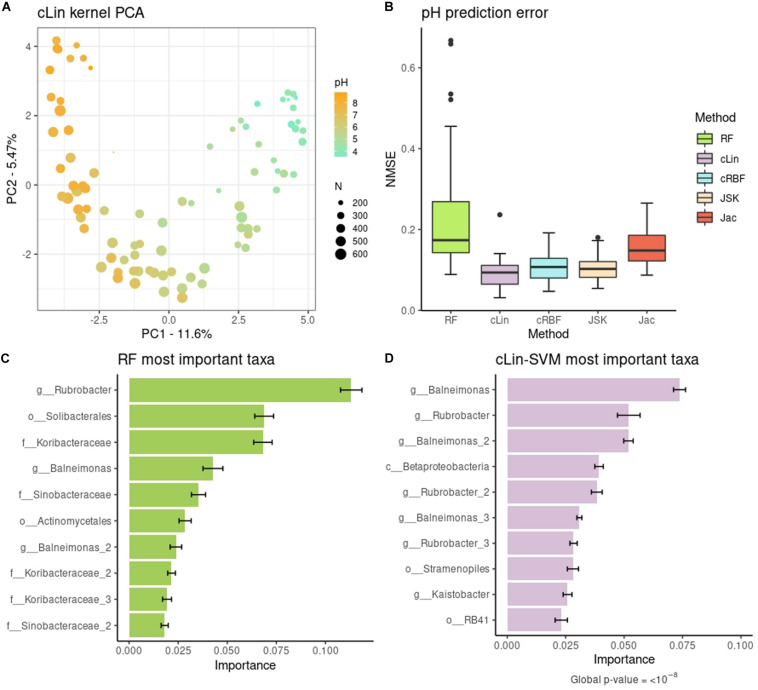
**(A)** Compositional linear kPCA over the 88 soils. Color represents pH, while point size stands for the number of different observed taxa. **(B)** pH prediction error distribution over the 40 replicates. **(C,D)** Top relevant taxa for pH prediction according to RF and cLin-SVM. Standard error across the 40 replicates is marked with error bars.

The cLin kPCA over the bacterial abundances is shown in Figure 2A. The remaining kPCAs, which gave a similar profile, can be found at Supplementary Figure 2. Soil samples are clearly separated by their pH, in agreement with the original results. The U-shaped projection is typical of data structured by a gradual transition with few overlapping OTUs at the endpoints (Supplementary Figure 3). The peak diversity in near-neutral soils in contrast with extreme pHs may also have some effect (Supplementary Figure 4). In addition, we used SVMs with the four kernels described above to predict the pH of each soil site from the bacterial abundances. This was not done in the original work and so we used RF, a non-kernel, alternative method, as benchmark. Results are shown in Figure 2B. The best compositional kernel was cLin, having a median error of ∼0.09; and the best non-compositional one was JSK, with a median error of ∼0.10. In comparison, RF had a higher median error, almost the double of cLin, around 0.17.

To go further in the interpretation of the results, we analyzed the microbial signature retrieved from RF and cLin-SVM. The distribution of the importances was highly skewed. For subsequent analyses we kept only 5% of the taxa, which accounted for around the 90% (RF) and 95% (SVM) of total importance, with the two methods having 42% of OTUs in common. Top ten relevant taxa are shown in Figure 2C (RF) and Figure 2D (SVM). In agreement with the kPCA results, prediction is primarily driven by few OTUs of extreme pH ecosystems (e.g., genera *Rubrobacter* and *Balneimonas* on the basic side, orders *Solibacterales* and *RB41* on the acid side). We used *MiRKAT* to test the significance of the association of the pH with both the top ten and 5% most important taxa, according to the cLin kernel. In both cases, we obtained very low *p*-values (<10^–8^).

### Smokers Data

**FIGURE 3:**
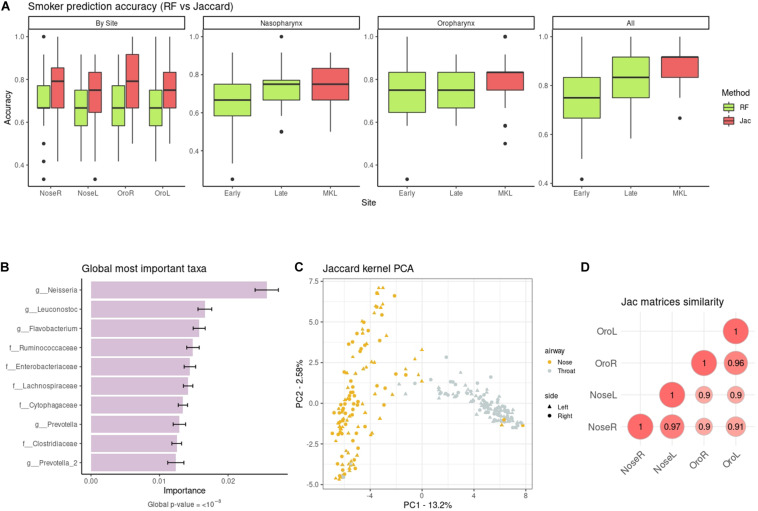
**(A)** Non-smoker/smoker accuracies from taxonomic data: RF (green) vs. the quantitative Jaccard kernel (red). NoseL, NoseR, OroL, and OroR models are obtained from single datasets, the Nasopharynx and Oropharynx panels contain the information from the right and left sides, and All the combination of the four datasets. Early and Late refer to the early integration and late integration approaches for RF. **(B)** Top ten global cLin-SVM importances across all body sites. **(C)** Similarity across the Jaccard kernel matrices of the four sites (Nasopharynx Right and Left and Oropharynx Right and Left). **(D)** Jaccard kPCA of the taxonomic abundances. Color code represent airway site, whereas shape indicates the laterality of the samples.

We predicted smoking status from the taxonomic abundances. At first models were built using the four sites separately, as in the original study. Authors used RF and reported a median accuracy of 64% on the right and 65% on the left oropharynx (i.e., throat), and 71% on the right and 68% on the left nasopharynx. We re-computed the RF accuracies with our data pre-processing, and obtained very similar results (Figure 3A), with the only exception of the right nasopharynx (new median accuracy: 66%). Regarding the kernels, the worst one was cLin (Supplementary Figure 5), which nonetheless gave similar accuracies to RF. The best kernel was the Jaccard kernel (Figure 3A), which improved substantially the RF accuracies, especially in the throat. Then, we combined the spatial-structured samples of the same individuals to test if accuracy increased when using an integrative approach (Figure 3A). For the kernels, we first used MKL to combine the kernel matrices at the airway level (nasopharynx on one hand and oropharynx on the other) and, finally, we integrated all sites. This decreased the error substantially and delivered the best classification result, with a median accuracy of 92%. As for the RF, we tested both the early integration approach and the late integration approach, and found that the latter granted better predictions. At best, integration of the four sites delivered a median accuracy of 83%. The results for the rest of kernels can be found in Supplementary Figure 5. In all cases, integration of the four datasets using our MKL proposal increased the accuracy in comparison to the individual models, and doing so gave better or equivalent results that those of RF integration approaches. The only exceptions to this trend were the nasopharynx and oropharynx models delivered by cLin (but not the model with the four sites combined).

Next, we recovered the overall microbial signature (i.e., across the four sampling sites). The importance distribution is not as skewed as in the Soil dataset: here the top 5% taxa accounted for the 62% of overall importance. The association of this subset of taxa with the target phenotype was highly significant (*p*-value < 10^–8^). Top ten taxa are shown in Figure 3B. *Neisseria* sp. large impact in discriminating smokers from non-smokers was already reported in the original work, especially in oropharynx models. The rest of highlighted taxa in Figure 3B were also noted to have a role, either in models from nasopharynx alone or from both airways sites (Charlson et al., 2010). This mostly agrees with our results when the sampling sites are analyzed separately (Supplementary Figure 6).

Following the original work, differences in bacterial communities among the body sites were also analyzed. We present results for the Jaccard kernel in Figures 3C,D, while the rest are in Supplementary Figure 7. Figure 3C shows the similarity across kernel matrices derived from left and right nasopharynx and oropharynx. The highest similarity was achieved within matrices of the same airway site but different laterality. As in the original paper (Supplementary Figure 8), using a kPCA (Figure 3D) we could discriminate between nasopharynx and oropharynx sites (first PC) but not between left and right.

### Pig Data

**FIGURE 4:**
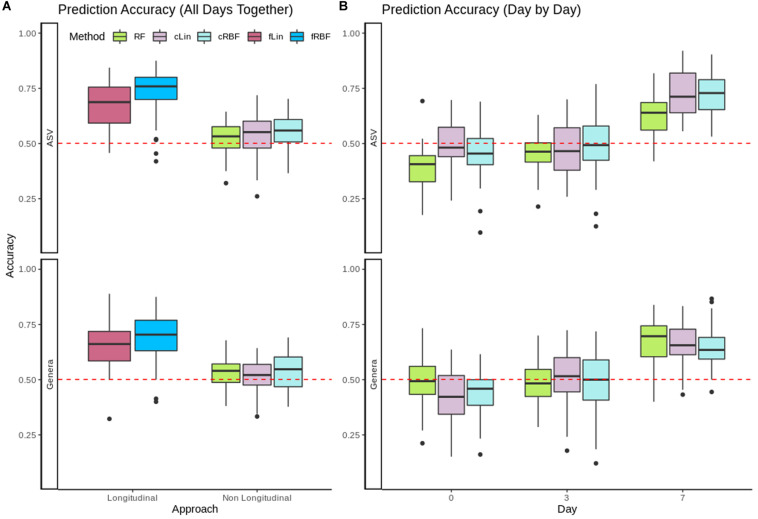
**(A)** Accuracy for RF, non-longitudinal kernels (cRBF, cLin) and Longitudinal kernels (fLin, fRBF) in the prediction of neonatal diarrhea from all available days. **(B)** Accuracy for RF and non-longitudinal cLin and cRBF kernels from metagenomic data of days 0, 3, and 7 post-birth separately. In both panels, the red dashed line marks the accuracy of the random model.

Evolution of gut microbiota from 153 healthy piglets over their first week of life was used to predict the occurrence of pre-weaning diarrhea. In Figure 4A we compared the performance of the longitudinal kernels (fLin and fRBF) vs. their analogous non-longitudinal kernels (cLin and cRBF) plus RF when using all available days at once. The longitudinal approach clearly outperformed the non-longitudinal approach at both Genera and ASVs levels. fRBF had a better performance than fLin, and worked best at the ASV level (with a median accuracy around 76%) than in Genera data (median accuracy ∼70%). Although aggregating taxa to the genus level is a relatively common practice –see e.g., Rivera-Pinto et al. (2018)–, in our case using a coarser taxonomic resolution decreased the accuracy. Within the non-longitudinal approach, we obtained similar accuracies using RF and kernels, and both were close to the median accuracy of the random model (50.1%). To further understand the results, the analysis was carried out in days 0, 3, and 7 separately using RF, cLin and cRBF kernels. Figure 4B reveals that all models from days 0 and 3 had no predictive power. Accuracy increased dramatically after day 7 to a maximum of 73% for cRBF (ASV level), only slightly worse than its analogous longitudinal kernel fRBF. We used the kernel machine test of *MiRKAT* to further confirm that days 0 and 3 were not significantly associated with phenotype, while day 7 was. As expected, only the kernel matrices of day 7 delivered significant *p*-values (Genera: cLin *p*-value < 10^–6^, RBF *p*-value < 10^–7^; ASV: cLin and cRBF *p*-values < 10^–8^) after Bonferroni correction.

**FIGURE 5:**
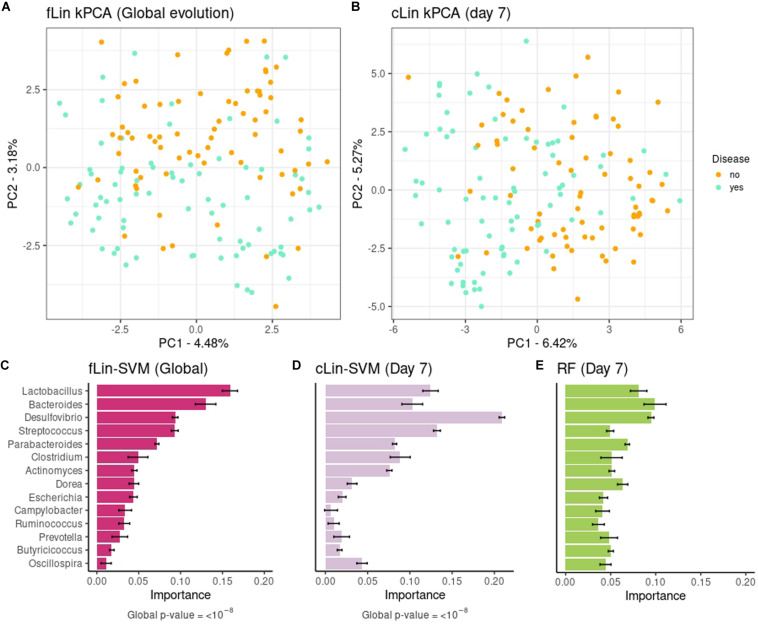
Above: kPCA of fLin in panel **(A)** and of cLin (day 7) in panel **(B)**. Below: Microbial signatures at the Genera level. Global importance for the first week is in panel **(C)**. Importances for the day 7 according to the cLin kernel and RF are in panels **(D,E)**. Standard error across the 40 replicates is marked with error bars.

In a second step we analyzed the kPCA and microbial signatures, after discarding all models without predictive power. Figures 5A,B show the fLin and cLin (day 7) kPCA, while fRBF and cRBF are in Supplementary Figure 9. In all cases a partial separation between healthy and sick piglets, with a large area of overlap, is observed. Genera relevance on prediction of pre-weaning diarrhea is shown in Figures 5C–E. We discuss the microbial signature at the Genera level, as around 2/3 of the ASVs lack species assignation (Supplementary Figure 10). According to fLin, beneficial genera like *Lactobacillus* and *Bacteroides* had the higher overall importance during the first week. In day 7, it was striking the great importance given by cLin to *Desulfovibrio*, and secondarily to *Streptococcus*. RF also highlighted the butyrate-producing genus *Dorea*. Distribution of the microbial signature at the ASV level was skewed, but again, much less than in the Soil case study. The top 5% ASVs accounted for a 46% (fLin) and 58% (cLin) of the total importance, with an overlap between RF and cLin in day 7 of 2/3 of the ASVs. The association of the 5% most important taxa (global and day 7) with the phenotype was statistically significant according to MiRKAT (*p*-values < 10^–8^).

## Discussion

The kernel framework allows performing a great diversity of analyses in a common ground, while allowing a great flexibility on how data is approached. However, within the microbiome field, previous application of kernel methods has been mostly restricted to specific areas. Zhan et al. (2017) proposed a kernel-based semi-parametric regression method for testing the association of the human microbiota communities with multiple phenotypes. Their method was implemented in the R package *MiRKAT*. In turn, Mariette et al. (2018) combined metagenomic data and environmental measures of the TARA ocean expedition using unsupervised MKL with the *mixKernel* package. In some reports that compare the performance of different supervised methods in microbiome data, SVM often appear along RF or ANN (Qu et al., 2019; Zhou and Gallins, 2019; Namkung, 2020). Thus, kernel methods were mostly used in an isolated way, without exploiting the kernel framework ability to integrate a great range of analyses while giving a unitary view. Another advantage of this framework is that it can handle virtually any data type. However, to our best knowledge, it has not been previously applied to longitudinal microbiome studies. Finally, in previous works there was a lack of kernels that took into account the compositional nature of metagenomic datasets. Here we addressed all these questions, while also providing some examples of how previous kernel-based tools like *MiRKAT* and *mixKernel* can fit into our framework.

When comparing *kernInt* to a popular package for microbiome analysis like QIIME2, it becomes apparent that the former is more specific in its scope. *kernInt* is not concerned with sequence alignment, taxonomic assignation and quality control as QIIME2 is, but with the analysis once the abundance table is obtained. Both packages are aimed at community ecology analysis (in QIIME2: alpha and beta diversities, PCoA, etc.) and supervised learning areas. While *kernInt* does not have the great range of methods available in QIIME2, it improves the current state-of-the-art in the following points: (i) Proposal and implementation of specific kernels for microbiota, while QIIME2 currently provides default kernels for real vectors (the linear, RBF, polynomial and sigmoid kernels). (ii) As far as we know, SVM is available in QIIME2 but kPCA is not; therefore, it is not possible performing both supervised and unsupervised analysis under the same mathematical point of view (Figure 1). (iii) Integration of spatial and temporal samples: QIIME2 does not have a specific handling of spatial (and, potentially, multi-omic) data, while *kernInt* allows performing unsupervised, supervised and retrieval of microbial signatures in this kind of datasets. On the other hand, the QIIME2 “longitudinal” plugin implements several tools for longitudinal data, but the option of performing supervised learning from the variation of microbiota over time is absent.

Throughout this work, we summarized the microbiome analyses in three branches: unsupervised learning (represented by kPCA), supervised learning (SVM) and identification of phenotype-associated microbial signatures. The Soil case study clearly illustrated how all three types are intertwined and complementary. In agreement with the original publication, both SVM and kPCA results showed that taxonomic abundances and pH are strongly related. This granted a quite low prediction error (up to a median NMSE of 0.09) but, by itself, does not explain the underlying mechanism connecting microbial abundance and pH. Microbial signature revealed that the SVM learning is driven by few taxa of opposite pH ecosystems. For instance, *RB41* belong to the phylum *Acidobacteria*. The *Rubrobacter* genus contains well known extremophiles and, like the *Balneimonas* (renamed *Microvirga*) genus, has preference for clearly alkaline soils (Dahal and Kim, 2017; Chen et al., 2018). Furthermore, the arch in the kPCA projection indicated that communities from acid and basic habitats did not overlap (Morton et al., 2017). Taken together, these complementary views point that soil microbial structure is shaped by a gradual niche differentiation strongly modulated by the pH. This agrees with previous findings on this dataset (Lauber et al., 2009; Morton et al., 2017) but appears in a more concise and unified way using the kernel framework.

In comparison to other methods, the kernel framework did not only allow a holistic view of data, but also gave good results in each learning area. Concerning supervised learning, in general, the kernel methods tend to have an advantage over variable-oriented methods (e.g., in supervised learning: ridge regression, decision trees, RF, etc.) and over ANN (for the reasons stated in the “Introduction” section) when faced with *N* < < < *D* data. This is a common scenario in metagenomics when working at the OTU or ASV level, but not necessarily in coarser taxonomic resolutions. This is illustrated with the different behavior of kernels with respect to RF in Figure 4B (see ASV vs. Genera results). In the other cases, SVM were consistently better (or at least equivalent) to RF in all the case studies that we analyzed. This disagrees with some previous reports in the microbiome area, e.g., Zhou and Gallins (2019). However, it should be noted that SVM performance depends on the kernel used, and these reports used generic linear and RBF kernels. Even when using kernels specific for metagenomic data, we observed differences among their mean NMSE or accuracies as large as fifteen percentage points. At the same time, our results suggest that there is not a single kernel that systematically achieves the best performance in every problem. We found that cLin was the best one in the first case study, quantitative Jaccard in the second and fRBF in the third. In this scenario, we consider that the linear-like kernels like cLin are a safe starting point. They allow for the retrieval of the microbial signatures, are faster to compute and easier to interpret than non-linear kernels, and with high-dimensional data (>10^3^–10^4^ taxa) they tend to match the RBF kernel (usually considered the gold standard) in performance (Hsu et al., 2003; Keerthi and Lin, 2003). RBF may be useful if the number of different taxa is low, or when a strong non-linear relationship is suspected. The weakness of the compositional kernels that we proposed is that they cannot handle zeroes without pre-processing; instead, zeroes pose no problems to the quantitative Jaccard kernel. How to deal with zeroes is, currently, an open topic of research in compositional analysis (Weiss et al., 2017; Quinn et al., 2018). If there is not enough *a priori* information that permits selecting the kernel beforehand, visual assessment of the candidate kernel matrices via kPCA could be of some help. A more rigorous approach is to perform nested cross-validation (Cawley and Talbot, 2010) to avoid overfitting when selecting both the candidate hyperparameters’ values and the best kernel for a given problem. Finally, phylogenetic kernels were beyond the scope of this work, nor the available datasets had the phylogenetic trees needed to compute them. However, they may be derived from (Eqs 1, 2) by replacing the clr term with other transformations, e.g., the PhILR transformation (Silverman et al., 2017). A phylogeny-based kernel was also proposed in Xiao and Chen (2017).

Concerning our unsupervised analyses, we observed that the main structure revealed by the original MDS/PCoA (ordination by pH in the Soil dataset, and by body site in the Smoker dataset) was conserved in our kPCAs. On the other hand, microbial signatures obtained with SVM had a biological interpretation. In general, the most important taxa retrieved from SVM coincided with those of RF (40–65% of overlap depending on the dataset), and could be recovered too when dealing with spatial and temporal-structured datasets. However, we acknowledge that a drawback of these signatures (though they handle well the cases of multicollinearity) is that they are based on linear kernels. In turn, RF can take into account both non-linearity and complex interactions among taxa. In any case, the informativeness of a microbial signature can be assessed by the prediction performance of the SVM model that generated it.

Apart of the aforementioned advantages of the kernel framework, we also showed how it can accommodate datasets with spatial and/or temporal components. We illustrated the integration of spatial-structured samples with the Smokers dataset. The analysis in the original work was carried out in each sampling site independently, with a maximum median accuracy of 71%. Here we showed how combining the body sites using MKL increased the median accuracy to 92%. Therefore, our results remark the relevance of using an integrative approach to improve the accuracy of phenotype prediction when spatial-structured samples of the same individuals are available.

In addition to the package and framework proposal, we analyzed a previously unpublished dataset profiling the microbiota evolution and pre-weaning diarrhea incidence in 153 piglets. Through this dataset we illustrated the kernel framework application to time-structured samples. Pre-weaning diarrhea is an important issue in pig production, as the antibiotic treatment increases both the emergence of resistances and the economic costs. It is already known that gut colonization starts immediately after birth, and it evolves from a highly variable to a more stable and homogeneous ecosystem over the first weeks. However, most of the current studies in pig production ignore early dynamics in gut microbiota (Mach et al., 2015; Han et al., 2018; Massacci et al., 2020). We wanted to test if pre-weaning diarrhea could be anticipated as soon as the first week of life. In this sense, our results suggest that the first stages of intestinal microbiota convey some valuable information indeed. kPCAs showed a partial separation between piglets affected of diarrhea vs. healthy piglets, and by using longitudinal kernels we achieved a moderate accuracy of 76%. However, it was unclear if this accuracy was to be attributed to a different taxa evolution in the two groups over the first week, or to a single time point with a great predictive value. The day-by-day prediction clarified this issue, and showed that day 7 achieved a median accuracy of 73% while the rest of points lacked predictive power. Even so, longitudinal kernels were able to slightly improve prediction (76% vs. 73% at the ASV level, and 69% vs. 64% using Genera), so global taxa evolution may also have a small role.

This is also seen in the underlying microbial signatures of the global first week (fLin) vs. day 7 (cLin). To be noted, in day 7 the most important genus was sulfate-reducing bacteria *Desulfovibrio*, which is known to have a relevant role during pig gut colonization (Mach et al., 2015). Instead, the global (longitudinal) model was mainly led by *Lactobacillus* and *Bacteroides*. Relationship of both genera to pre-weaning diarrhea is well sustained in literature. *Lactobacillus* spp. are well known probiotic bacteria, while members of *Bacteroides* genus are associated with increased infants gut microbial diversity (Stewart et al., 2018). Furthermore, both play an important role on mammals’ gut microbial colonization (Sawicki et al., 2017; Wexler and Goodman, 2017) and are dominant in healthy pigs compared with diarrhea-affected piglets (Song et al., 2017), which gives confidence in the reliability of our findings.

In summary, our kernel framework successfully places the most important analyses in the microbiome field on a common ground, takes into account the compositionality of data, and is flexible enough to integrate spatial and temporal dimensions of the datasets.

## Data Availability Statement

The data analyzed in this study is subject to the following licenses/restrictions: This manuscript utilizes proprietary data. Requests to access these datasets should be directed to YR-C/IRTA/yuliaxis.ramayo@irta.cat.

## Ethics Statement

The animal study was reviewed and approved by the Central Authority for Scientific Procedures on Animals of Netherlands–Centrale Commissie Dierproeven (CCD).

## Author Contributions

YR-C, MP-E, RQ, and ER contributed to conception and design of the study. FM was in charge of the pig data sampling. YR-C and MP-E supervised the overall research, while LB-M supervised the machine learning part. ER performed the all analysis and wrote the first draft of the manuscript. YR-C, MP-E, and LB-M revised and wrote sections of the manuscript. All authors contributed to manuscript revision, read, and approved the submitted version.

## Conflict of Interest

The authors declare that the research was conducted in the absence of any commercial or financial relationships that could be construed as a potential conflict of interest.

## Abbreviations

ANN: artificial neural network
ASV: amplicon sequence variant
JSK: Jensen-Shannon Kernel
kPCA: kernel principal components analysis
MDS: multidimensional scaling
MKL: multiple kernel learning
NGS: next-generation sequencing
NMSE: normalized mean squared error
OTU: operational taxonomic unit
PCA: principal components analysis
PCoA: principal coordinates analysis
RBF: radial basis function
RF: random forests
SVM: support vector machines
