# Supplementary material for: kernInt: A Kernel Framework for Integrating Supervised and Unsupervised Analyses in Spatio-Temporal Metagenomic Datasets
Source: Front Microbiol. 2021 Jan 28;12:609048. doi: 10.3389/fmicb.2021.609048 (PMC7876079; doi:10.3389/fmicb.2021.609048)
Supplement: Supplementary file 1 [file Data_Sheet_1.pdf]

Supplementary Material

1 Supplementary Method 0

The experiment was conducted at Schothorst Feed Research B.V. facilities where management, environmental and housing factors were controlled throughout the whole study. All animals were born to Topigs-Norsvin 70 (TN70) sows. Sampling (swabs) was done for healthy piglets across three age strata: within 5 minutes after farrowing (i.e. day 0) and at days 3 and 7 post-farrowing. DNA from fecal samples was extracted at IRTA laboratory with the DNeasy PowerSoil Kit (QIAGEN). Extracted DNA was sent to the University of Illinois Keck Center for Fluidigm sample preparation and Illumina sequencing of 16S rRNA gene. Primers targeting the V3 – V4 region (F357 and -R805) were used to amplify a region of 552 base pairs of the bacterial 16S rRNA gene and sequenced on one MiSeq flowcell for 251 cycles. Sequences corresponding to the V3-V4 region of the 16S rRNA gene were analysed using QIIME2 software (Bolyen et al. 2018). The workflow included a quality control step to remove sequences with Phred scores of < 30, trim sequences based on expected amplicon length, remove chimera and merge paired reads. The cleaned 16S rRNA gene sequences were processed into Amplicon Sequences Variants (ASVs) and classified to the lowest possible taxonomic rank using QIIME2 (Bolyen et al 2018) against the GreenGenes Database release 2013-08 (DeSantis et al. 2006). Moreover, samples with less than 900 reads were excluded and not considered in posteriors analysis. A total of 153 piglets and 3.29·106 reads were retained for subsequent data analyses (Figure 2) after filtering out the low quality reads and samples with less the 900 reads. As expected, all negative control samples (42) were excluded in the quality control, which support the quality of DNA extraction and sequencing processes. Due to the low DNA concentration and the low bacterial biomass some samples from piglets' microbiota at day 0 did no pass the filtering process. In the final step of the quality control we also filtered out singletons and doubletons, to finally obtain a total of 3,577 ASVs.

2 Supplementary Tables

Table 1. Candidate hyperparameters' ranges

	Soil	Smoker	Pig
**Number of trees (RF)**	500, 750, 1000, 2000, 3000		
**C (SVM)**	1, 10, 25, 50, 100		
**ε (SVM)**	0.01	-	-
**γ (cRBF)**	$10^{-5}, 10^{-4}, 10^{-3}, 10^{-2}$		$10^{-4}, 10^{-3}, 10^{-2}, 10^{-1}$
**γ (fRBF)**			$10^{-6}, 10^{-5}, 10^{-4}, 10^{-3}$
**MKL β coefficients (2 sites)**		{0.5,0.5}, {0.25,0.75}, {0.75,0.25}	
**MKL β coefficients (4 sites)**		{0.25,0.25,0.25,0.25}, {0.35,0.35,0.15,0.15}, {0.15,0.15,0.35,0.35}	

3 Supplementary Figures

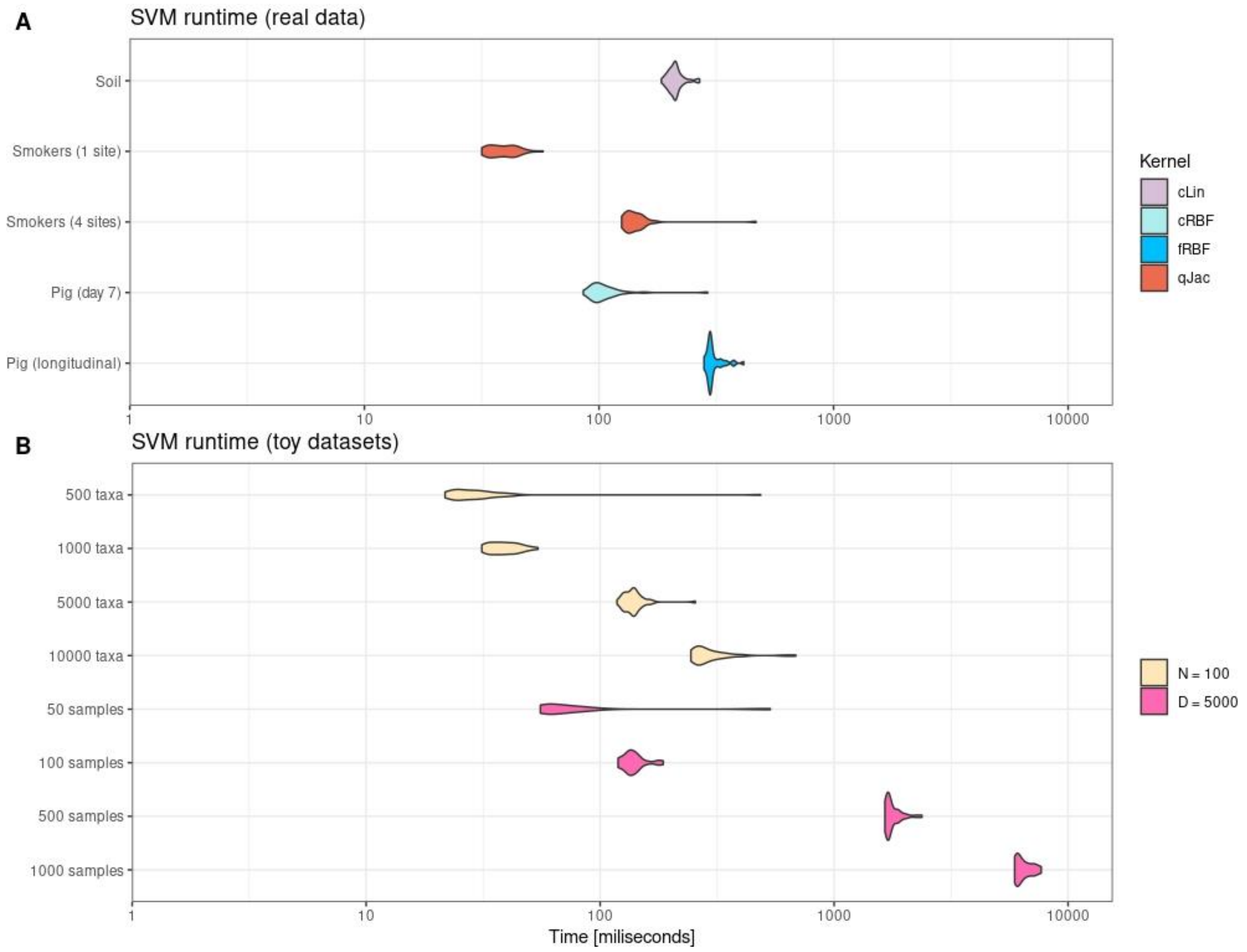

Supplementary Figure 1. Running time of computing a single SVM model from the Soil, Smokers and Pig datasets (panel A) and from synthetic count data (panel B). The analysis was run 40 times to obtain a time distribution. In panel A, we show the best-performing kernel and cost (C) value for each problem (see Results section). In panel B, we evaluated a classification problem with C=1, and the cLin kernel was used in all instances.

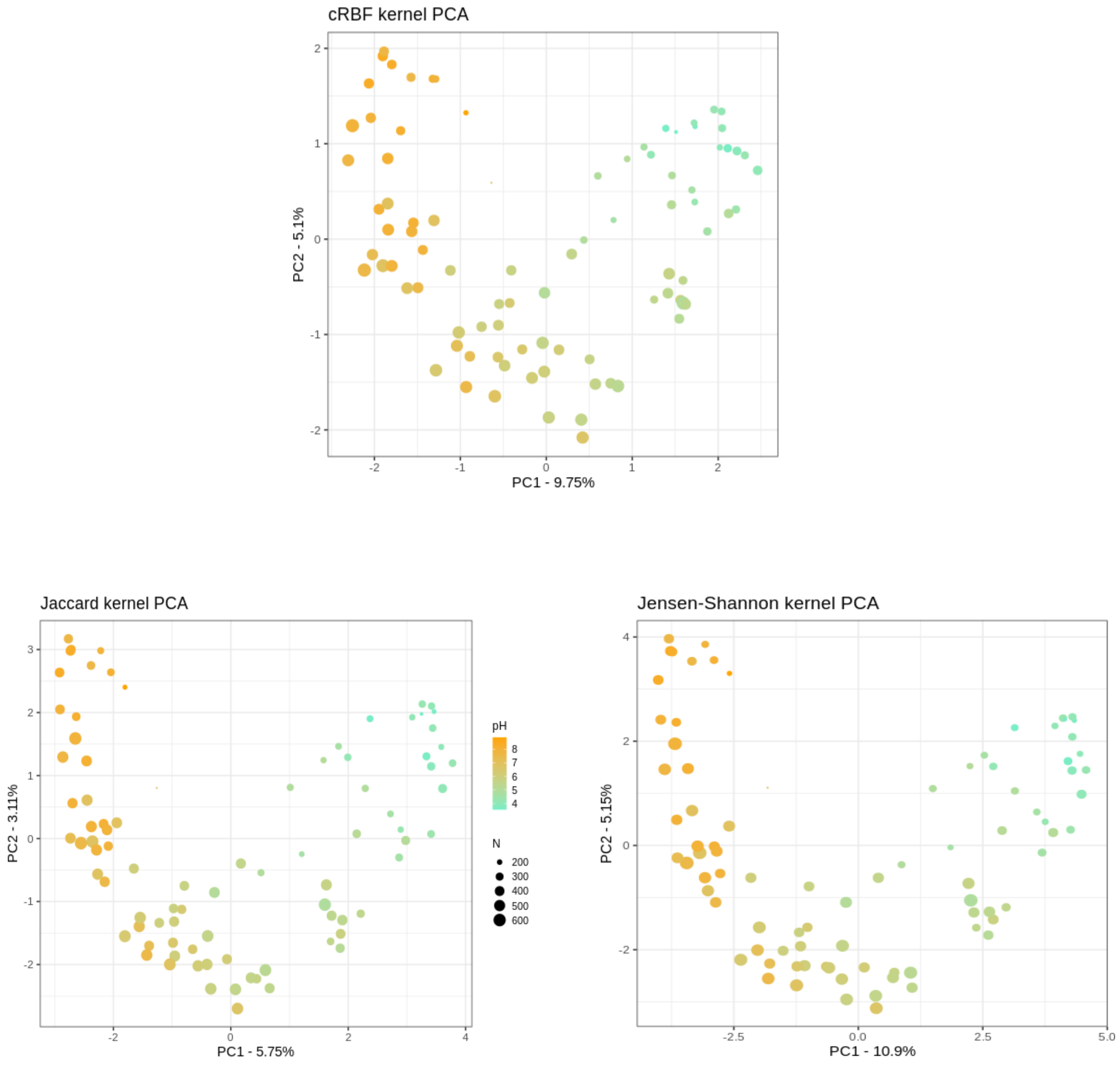

Supplementary Figure 2. Soil kPCAs for the cRBF kernel, JSK and the Jaccard kernel. Same legend than in Figure 1A.

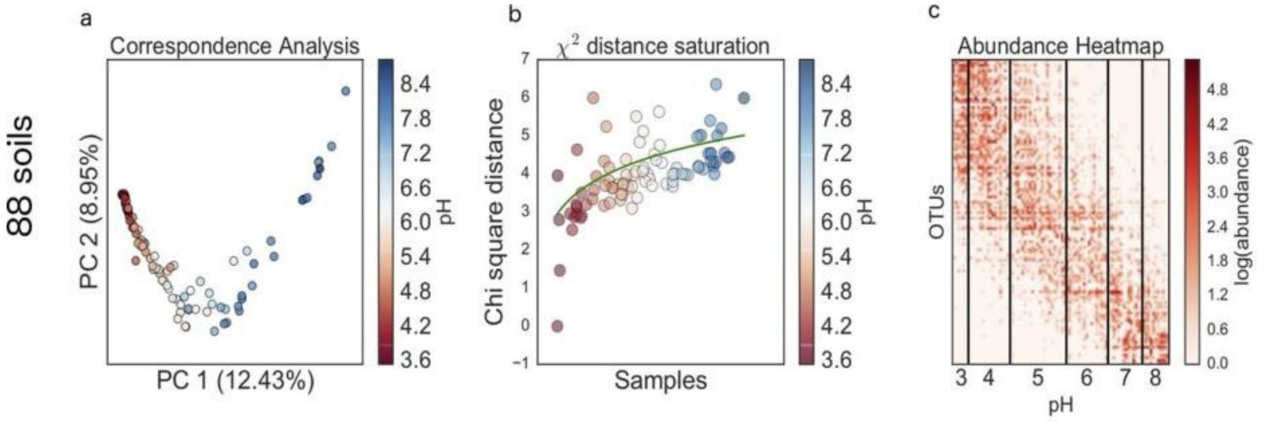

Supplementary Figure 3. Morton et al. (2017) revisit Lauber data. The Correspondence Analysis in panel a show a clear U-shaped effect. Panel c demonstrates the band nature of the Soil dataset.

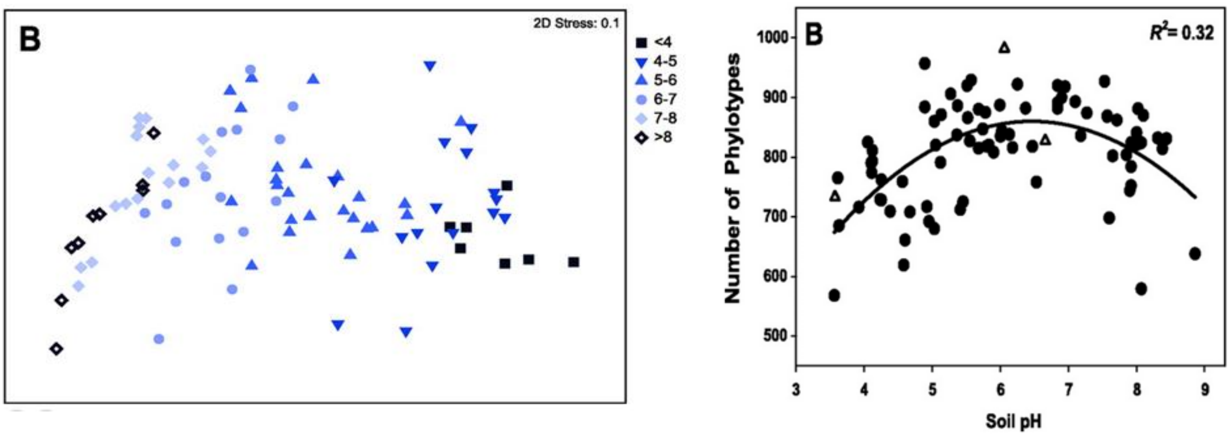

Supplementary Figure 4. Original results by Lauber et al. (2009). Left: MDS plot derived from Unifrac distances with shape indicating soil pH. The arch pattern is visible but less steep than in Figure 1A, S1 and S2 because of the coarser taxonomic resolution in the original study (Morton et al., 2017). Right: soil pH correlation to number of phylotypes.

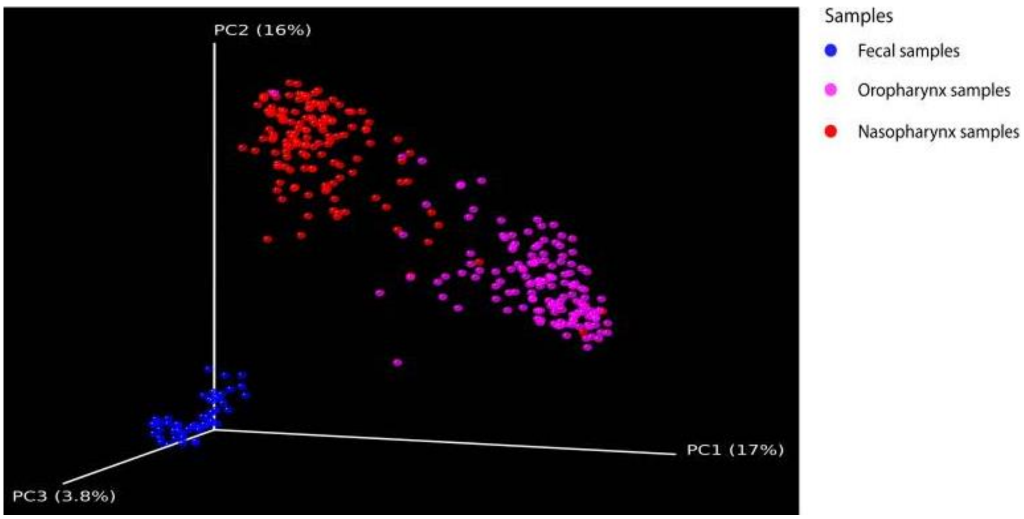

Supplementary Figure 5. PCoA of the taxonomic abundances reported in the original paper by Charlson et al. (2010). Colors denote body sites: oropharynx (red), nasopharynx (pink) and fecal (blue).

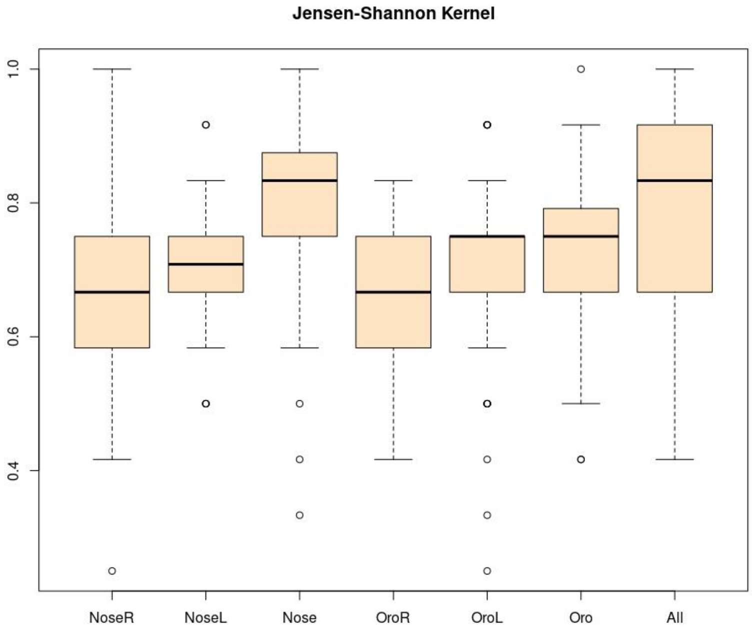

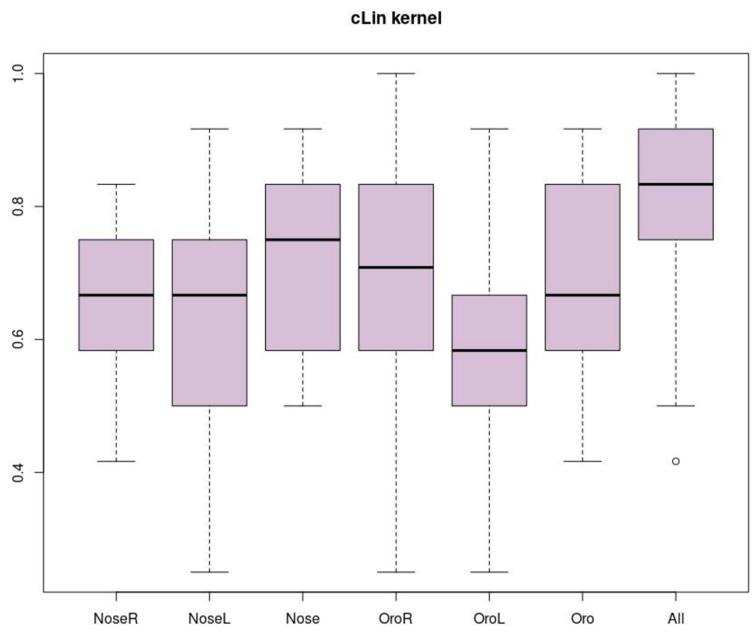

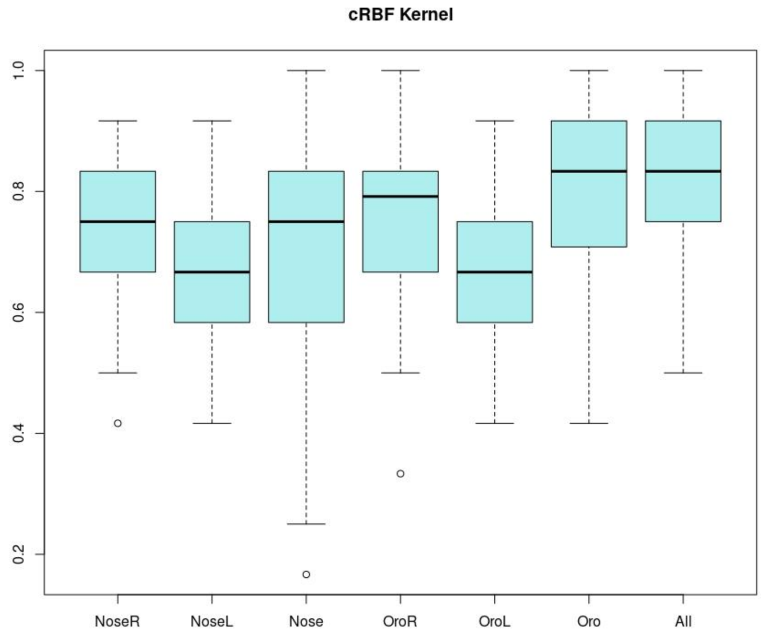

Supplementary Figure 6. Smoker/nonsmoker prediction accuracy using JSK, cLin and cRBF + SVM. ‘Nose’, ‘Oro’ and ‘All’ models were obtained using MKL.

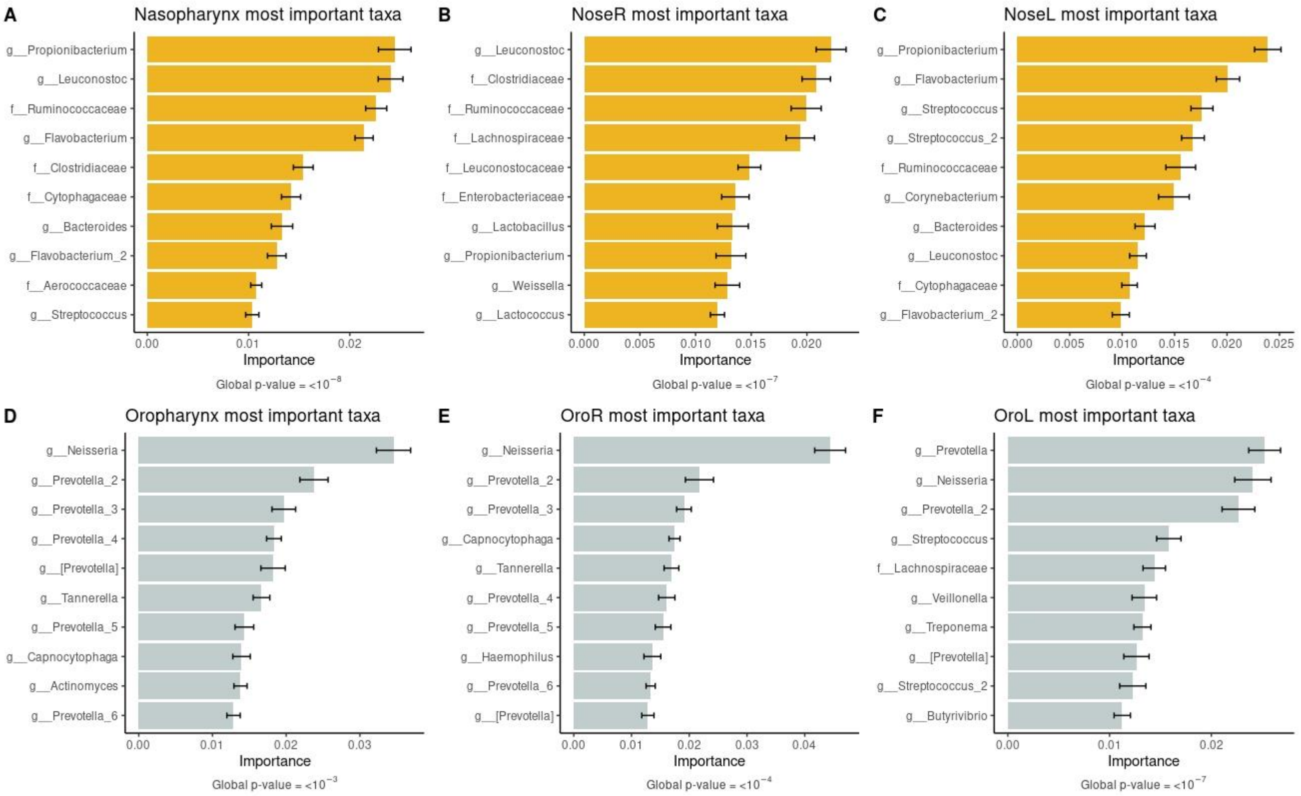

Supplementary Figure 7. Top ten relevant taxa for the nasopharynx (A) and oropharynx (D) MKL models, and for the four body sampling sites separately (B, C, E, F). p-values computed with the *MiRKAT* package.

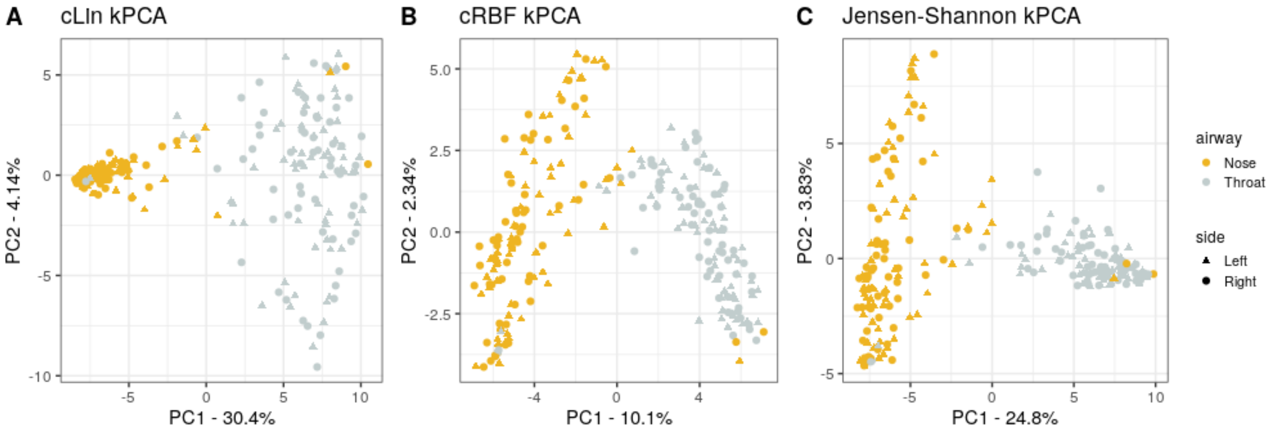

Supplementary Figure 8. cLin, cRBF and Jensen-Shannon kernel PCA. Same legend than Figure 2D.

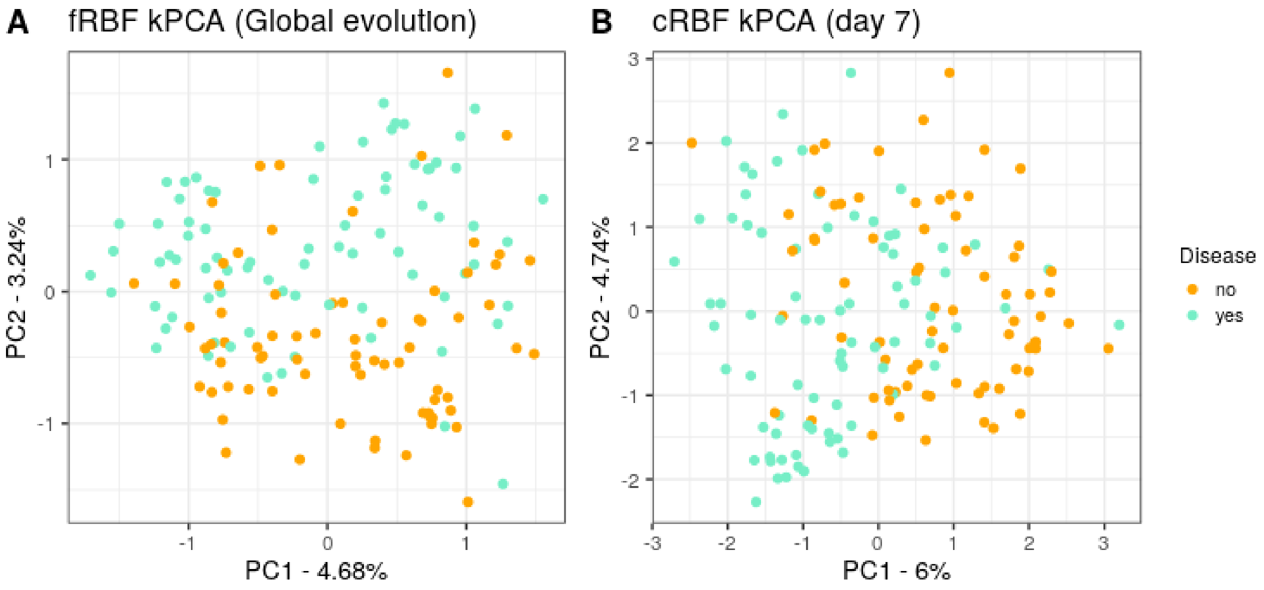

Supplementary Figure 9. fRBF and cRBF kernel PCA for the ASV data. Same legend than Figure 4 A and B.

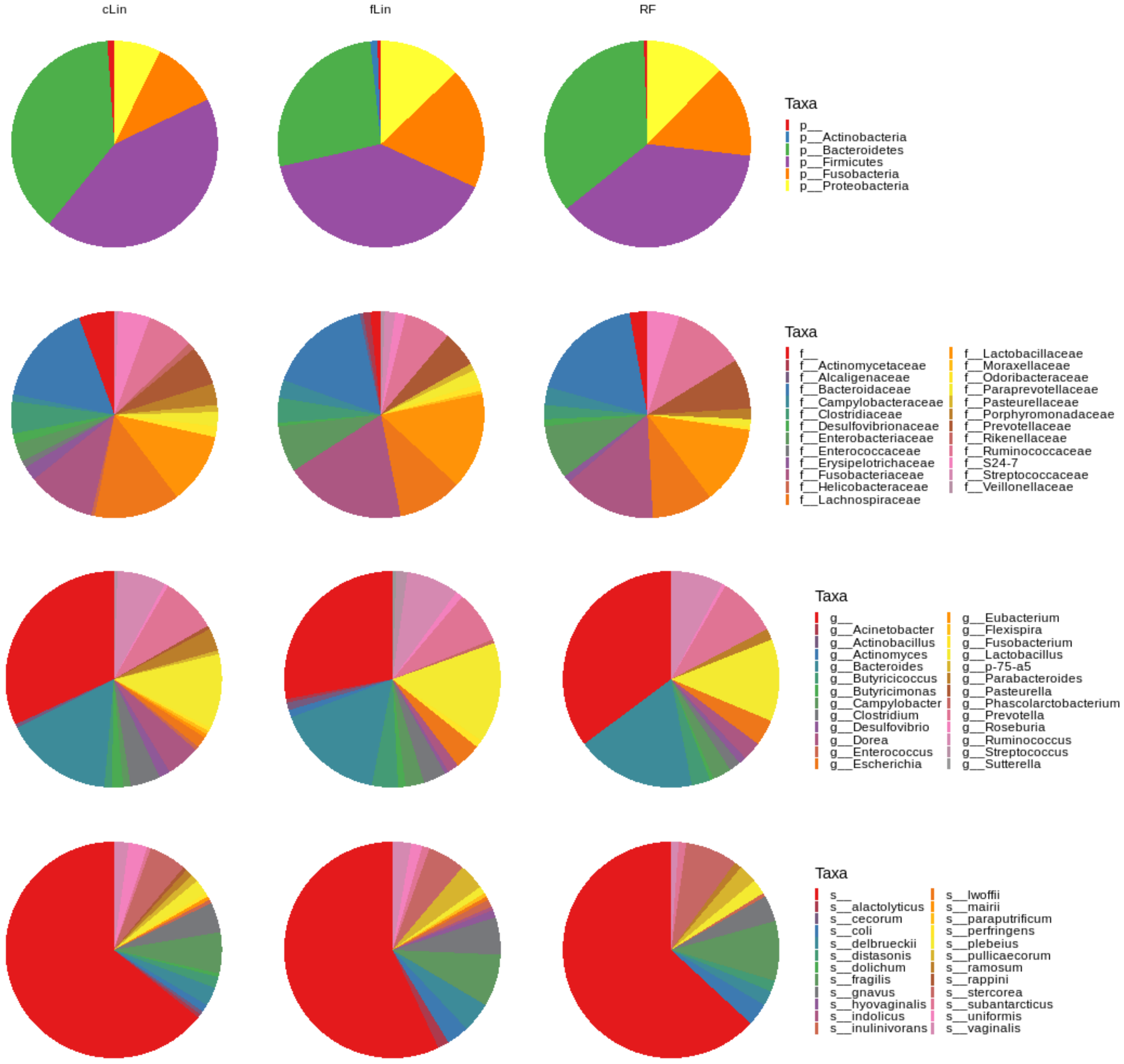

Supplementary Figure 10. Importance distribution at the Phyla, Family, Genera and Species level of the top 5%: cLin and RF (day 7) and fLin (global).
